# MRI-based radiomics analysis for preoperative evaluation of lymph node metastasis in hypopharyngeal squamous cell carcinoma

**DOI:** 10.3389/fonc.2022.936040

**Published:** 2022-09-23

**Authors:** Shanhong Lu, Hang Ling, Juan Chen, Lei Tan, Yan Gao, Huayu Li, Pingqing Tan, Donghai Huang, Xin Zhang, Yong Liu, Yitao Mao, Yuanzheng Qiu

**Affiliations:** ^1^ Department of Otolaryngology-Head and Neck Surgery, Xiangya Hospital, Central South University, Changsha, China; ^2^ Otolaryngology Major Disease Research Key Laboratory of Hunan Province, Xiangya Hospital, Central South University, Changsha, China; ^3^ Clinical Research Center for Pharyngolaryngeal Diseases and Voice Disorders in Hunan Province, Xiangya Hospital, Central South University, Changsha, China; ^4^ National Clinical Research Center for Geriatric Disorders, Xiangya Hospital, Central South University, Changsha, China; ^5^ College of Computer and Information Engineering, Hunan University of Technology and Business, Changsha, China; ^6^ Department of Head and Neck Surgery, Hunan Cancer Hospital, Xiangya Medical School, Central South University, Changsha, China; ^7^ Department of Radiology, Xiangya Hospital, Central South University, Changsha, China

**Keywords:** hypopharyngeal squamous cell carcinoma, lymph node metastasis, prediction model, magnetic resonance imaging, radiomics

## Abstract

**Objective:**

To investigate the role of pre-treatment magnetic resonance imaging (MRI) radiomics for the preoperative prediction of lymph node (LN) metastasis in patients with hypopharyngeal squamous cell carcinoma (HPSCC).

**Methods:**

A total of 155 patients with HPSCC were eligibly enrolled from single institution. Radiomics features were extracted from contrast-enhanced axial T-1 weighted (CE-T1WI) sequence. The most relevant features of LN metastasis were selected by the least absolute shrinkage and selection operator (LASSO) method. Univariate and multivariate logistic regression analysis was adopted to determine the independent clinical risk factors. Three models were constructed to predict the LN metastasis status: one using radiomics only, one using clinical factors only, and the other one combined radiomics and clinical factors. Receiver operating characteristic (ROC) curves and calibration curve were used to evaluate the discrimination and the accuracy of the models, respectively. The performances were tested by an internal validation cohort (n=47). The clinical utility of the models was assessed by decision curve analysis.

**Results:**

The nomogram consisted of radiomics scores and the MRI-reported LN status showed satisfactory discrimination in the training and validation cohorts with AUCs of 0.906 (95% CI, 0.840 to 0.972) and 0.853 (95% CI, 0.739 to 0.966), respectively. The nomogram, i.e., the combined model, outperformed the radiomics and MRI-reported LN status in both discrimination and clinical usefulness.

**Conclusions:**

The MRI-based radiomics nomogram holds promise for individual and non-invasive prediction of LN metastasis in patients with HPSCC.

## Introduction

Hypopharyngeal squamous cell carcinoma (HPSCC) is a rare type of head and neck cancer with an estimated 5-year overall survival rate of 30–35% (1, 2). As early symptoms are relatively atypical, tumors often progress to advanced stages when diagnosed, while more than 50% of patients presented with neck lymph node metastasis (3). The current American Joint Committee on Cancer (AJCC) staging system has placed weight on the size, laterality and extra-nodal extension of lymph nodes for HPSCC. Lymph node metastasis is an independent prognostic factor for HPSCC, and patients with pathological cervical nodal metastasis have a significantly higher risk of recurrence (4, 5). For patients with suspected lymph node metastasis clinically, neck dissection was recommended by National Comprehensive Cancer Network (NCCN) guideline. Hence, to avoid unnecessary neck dissection and its possible complications, accurate prediction of lymph node metastasis in HPSCC is crucial for clinical decision-making and the improvement of prognosis.

Imaging tools are widely used in clinical for determining nodal status because of their non-invasive properties and high accuracies (6). Radiologist and oncologist using imaging tools to assess clinical lymph nodes staging mainly based on the size and shape of the lymph nodes. However, reactive or inflammatory lymph nodes can be enlarged and metastatic lymph nodes can also be normally shaped (7, 8). Hence, a proportion of patients are at high risk for inaccurate clinical nodal staging. Radiomics is a rapidly developing field that converts medical images into high-dimensional, mineable data *via* high-throughput extraction of quantitative features (9, 10), which has been widely used in developing oncological diagnosis and prognosis biomarkers (11, 12). Recently, Radiomics features extracted from primary tumors exhibited great potential in prediction of cervical lymph node metastasis (13, 14). However, only a few studies have explored the role of radiomics features in lymph node evaluation of head and neck cancers (15, 16). To our knowledge, there is no radiomics study for prediction of cervical lymph node metastasis in HPSCC.

The purpose of this study was to investigate the value of radiomics features extracted from contrast-enhanced MRI, combined with clinical information, for the preoperative prediction of lymph node metastasis in patients with HPSCC.

## Materials and methods

### Patients

This retrospective study was approved by the Medical Ethics Committee of Xiangya Hospital (IRB: NO2019121179), and written informed consent was waived. Patient cohorts were collected from September 2009 to September 2021 in Xiangya Hospital with the following inclusion criteria: (1). Histopathology confirmed as HPSCC with definite postoperative pathologic lymph node status; (2). Accessible pretreatment contrast-enhanced MRI sequence; (3). Intact clinicopathologic data and follow-up information; (4). Primary tumor > 1cm in greatest dimension. The exclusion criteria were as follows: (1). Patients received chemotherapy or radiotherapy before surgery; (2). Lack of definite result of postoperative lymph node status; (3). Corrupted MRI images or any visible artefacts in tumor area; (4). Primary tumor ≤ 1cm in greatest dimension. In consequence, a total of 155 patients were enrolled and randomly divided (7:3) into a training and an internal validation cohort.

The baseline clinical and histopathological data were recorded in detail from medical documents, including age, smoking, alcohol consumption and the sites of the primary lesions. MRI data of the patients were acquired from the Picture Archiving and Communication System (PACS).

### Image acquisition, segmentation and MRI-reported LN status

All Patients underwent a pretreatment 1.5T MRI scan performed on multiple MRI scanners (MAGNETOM Area, Siemens Healthineers AG) with the same protocol [420 msec repetition time (TR); 11 msec echo time (TE); 5mm slice spacing; 1.0mm intersection gap; 24 × 24cm field of view (FOV); 0.8594 × 0.8594mm pixel spacing; 4mm slice thickness;150 degrees flip angle and 256 × 256 matrix]. MRI Digital Imaging and Communications in Medicine (DICOM) images were archived from PACS. Contrast-enhanced axial T1 weighted (CE-T1WI) DICOM images were collected for segmentation in this study. Tumor area of HPSCC was defined as region of interest (ROI) and manually drawn slice by slice using ITK-SNAP software (v.3.8.0, www.itksnap.org) on CE-T1WI images, which were delineated by one senior radiologist with 10 years of experience in head and neck cancer and confirmed by a professor with more than 20 years working experience.

Two experienced radiologists reviewed the MRI images to evaluate the status of lymph nodes in consensus blinded to the pathological information. Any disagreement was resolved by consultation.

### Feature extraction and radiomics model development

**Figure 1:**
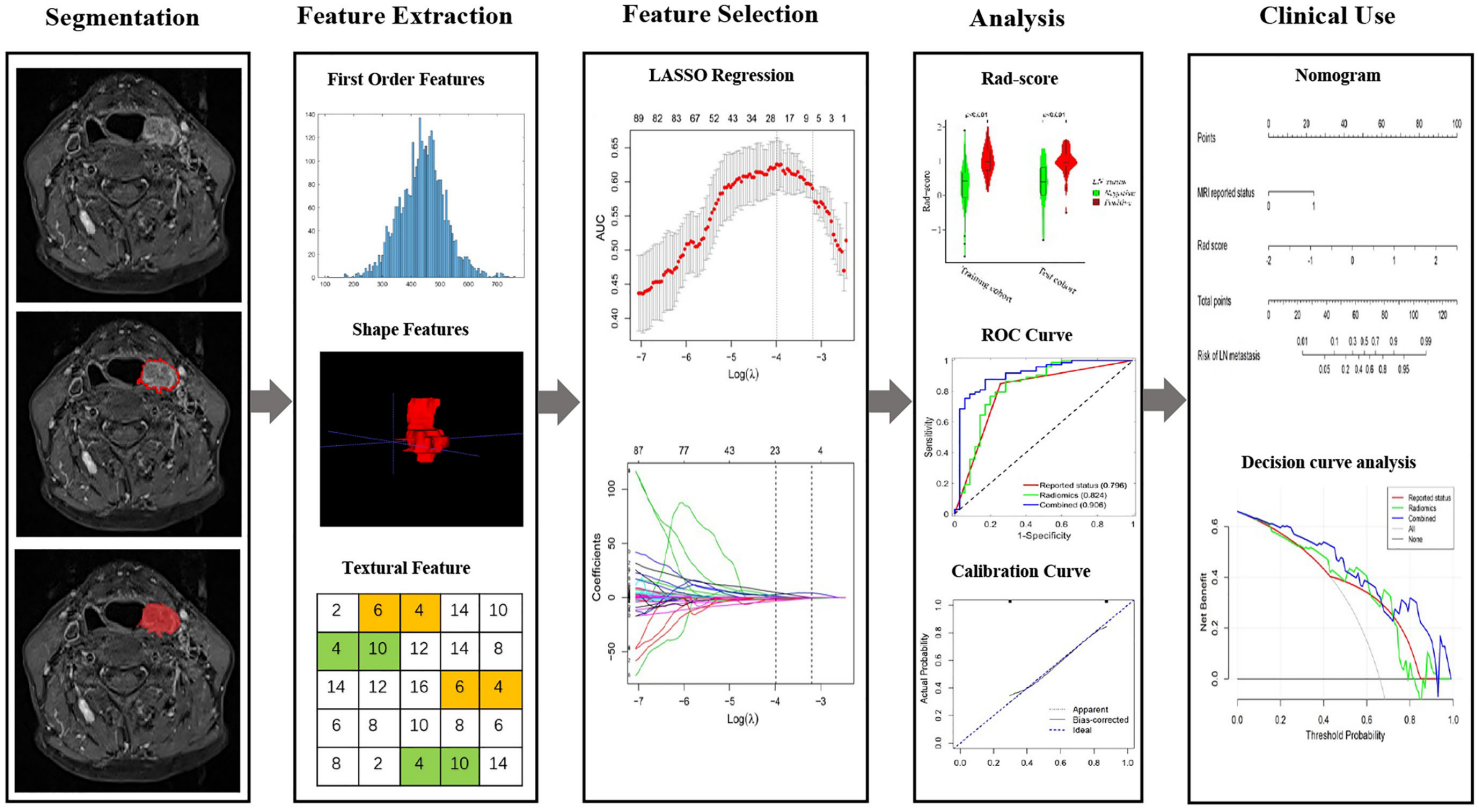
The flowchart of this study. Tumor segmentation was performed on Contrast-enhanced axial T-1 weighted MR images. Experienced otolaryngology head and neck surgeon contoured the tumor areas on MRI slices. Radiomic features to quantify tumor intensity, shape and texture were extracted from original MR data. The LASSO regression was used to select features. The rad-score is constructed by a linear combination of selected features. The performance of the prediction model is assessed by the area under a receiver operating characteristic (ROC) curve and the calibration curve. Radiomics nomogram was established and the calibration curve was used to evaluate the established model.

The flowchart of radiomics model construction was presented in **Figure 1**. As we described before (17, 18), Z-score normalization was applied as a preprocessing step, and 530 radiomics features were extracted with PyRadiomics (https://pyradiomics.readthedocs.io/en/latest/), including 18 first-order gray features, 8 shape features, 24 gray co-occurrence matrices (GLCMs), 16 gray run-length matrices (GLRLMs) and 8 sets of wavelets transform features, while each set of features had 18 first-order gray features, 24 GLCMs and 16 GLRLMs. The radiomics features were calculated based on a 3D ROI. All these features were extracted with the default setting in PyRadiomics, which guarantees the most consistency with the Image Biomarker Standardization Initiative (IBSI) guidelines (https://pyradiomics.readthedocs.io/en/latest/faq.html). Inter-class correlation coefficients (ICC) were applied to evaluate the inter-reader agreement, with ICC < 0.50 indicating poor agreement, 0.50 ≤ ICC < 0.75 moderate agreement, 0.75 ≤ ICC < 0.90 good agreement, and ICC ≥ 0.90 excellent agreement.

Images from patients in training cohort were used for feature selection and radiomics model development. The least absolute shrinkage and selection operator (LASSO) conducted with 8-fold cross-validations was applied to select the most significant features and construct the formula for radiomics score (Rad-score), Rad-score of each patient was calculated accordingly (Rad score = intercept + feature 1 × coefficient 1 + feature 2 × coefficient 2…), 22 features were selected and applied for Rad-score calculation.

### Radiomics nomogram construction and validation

Univariate and multivariate logistic regression analysis were performed to determine the potential independent risk factors. Then according to the result of multivariate analysis, a clinical-radiomics nomogram that integrated the radiomics signature and the independent clinicopathological risk factors was constructed to predict LN metastasis status. Meanwhile, radiomics signature and the independent clinicopathological risk factors independently constructed models, respectively.

AUCs were calculated in training and validation cohort to quantify the discrimination performance of the models. The accuracies of the models were assessed with a calibration curve. The nomogram of the combined model was presented as an intuitive tool for clinicians to assess the risk of LN metastasis in patients with HPSCC. The clinical usefulness of the models was assessed by quantifying the net benefits at different threshold probabilities determined by the decision curve analysis.

### Statistical analysis

All the statistical analysis were performed with R software (version 3.6.1; http://www.R-project.org). Differences between the two groups were compared using a *t*-test, Pearson’s chi-square test or Fisher’s exact test. Delong test was used to compare the ROC curves of different models, and Bonferroni correction was applied since multiple comparison was involved. The following R packages were used: “glmnet” implements the LASSO method; “rms” constructs the nomogram and implements the calibration curve analysis; “rmda” implements the decision curve analysis; “broom” tidies the data or modifies the format of the data; and “ggplot2” draws the violin plot and polishes the graphs. All statistical tests were two-sided with a statistical significance of *P* < 0.05.

## Results

### Clinical characteristics

A total of 155 eligible patients were finally enrolled randomly divided (with a ratio of 7:3) into a training cohort (n=108 [69.7%]) and a validation cohort (n=47 [30.3%]). Clinical characteristics of the patients in the training and validation cohort are showed in **Table 1**. No significant differences were observed between the training and validation cohorts in terms of age, tobacco use, alcohol consumption, tumor sites and MRI-reported LN status. In our study, 67.6% (73/108) and 66.0% (31/47) patients had LN metastasis in the training and validation cohort, respectively. The accuracy of routine MRI in the diagnosis of LN metastasis was 78.7%.

**Table 1:** Characteristics of patients in training and validation cohorts.

Characteristic	Training cohort			Validation cohort			
	LNM (+)(73,68%)	LNM (-)(35,32%)	P	LNM (+)(31,66%)	LNM (-)(16,34%)	P	P^#^
**Age (years, mean± SD**)	59.44 ± 9.07	58.20 ± 9.62	0.516	59.35 ± 8.69	57.31 ± 11.00	0.490	0.817
**Tobacco use**			0.912			0.112	0.122
Yes	57 (52.8%)	27 (25.0%)		18 (38.3%)	13 (27.7%)		
No	16 (14.8%)	8 (7.4%)		13 (27.7%)	3 (6.4%)		
**Alcohol consumption**			0.662			0.357	0.028
Yes	57 (52.8%)	26 (24.1%)		17 (36.2%)	11 (23.4%)		
No	16 (14.8%)	9 (8.3%)		14 (29.8%)	5 (10.6%)		
**Location**			0.659			0.496	0.125
Pyriform sinus	51 (47.2%)	22 (20.4%)		23 (48.9%)	14 (29.8%)		
Posterior pharyngeal wall	20 (18.5%)	11 (10.2%)		8 (17.0%)	2 (4.3%)		
Post cricoid	2 (1.9%)	2 (1.9%)		–	–		
**MRI-reported LN status**			< 0.001			0.003	0.218
LNM (+)	62 (57.4%)	9 (8.3%)		22 (46.8%)	4 (8.5%)		
LNM (-)	11 (10.2%)	26 (24.1%)		9 (19.1%)	12 (25.5%)		

P value was derived from the univariable association analyses between each of the clinical characteristics and lymph node status. P^#^ represented the difference of each clinical characteristic between the training and validation cohorts.

LNM (+), positive lymph node metastasis; LNM (-), negative lymph node metastasis; MRI-reported LN status, Magnetic resonance imaging- reported lymph node status.

### Feature selection and radiomics model construction

**Figure 2:**
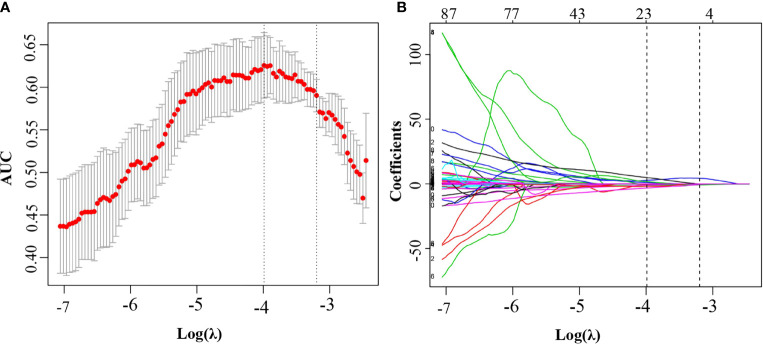
Feature selection using the least absolute shrinkage and selection operator regression model. **(A)** Selection of tuning parameter (λ) in the LASSO model used 8-fold cross-validation *via* minimum criteria. The area under the receiver operating characteristic (AUC) curve was plotted versus log(λ). Dotted vertical lines were drawn at the optimal values by using the minimum criteria and the 1 standard error of the minimum criteria (the 1-SE criteria). The optimal λ value of 0.019 with log (λ) of −3.988 was chosen. **(B)** LASSO coefficient profiles of the 530 selected features. A coefficient profile plot was produced against the log (λ) sequence. A vertical line was plotted at the optimal λ value, which resulted in 22 features with nonzero coefficients.

**Figure 3:**
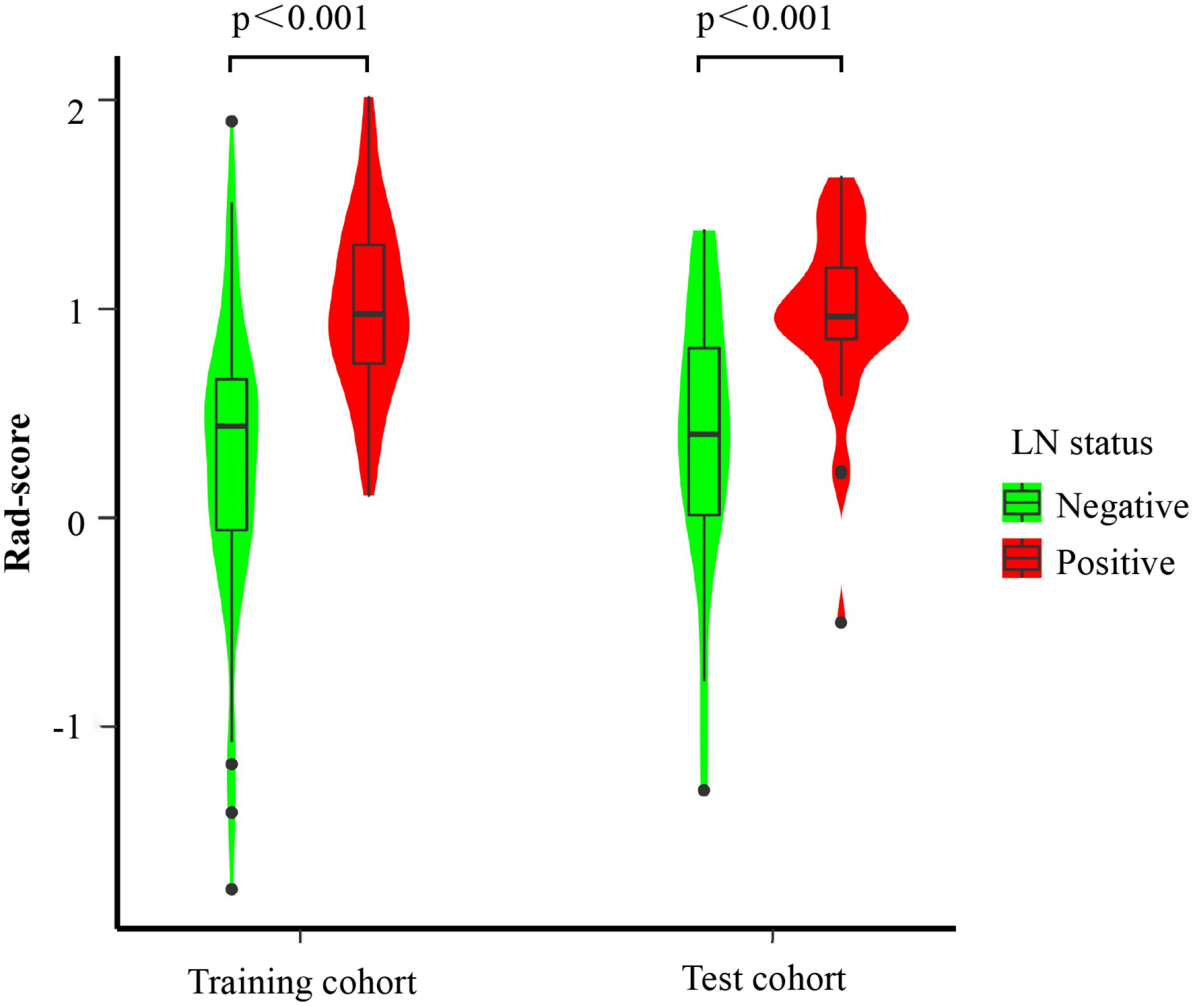
Boxplots of the Radiomics score (Rad-score) for the training and validation cohort.

Among the 530 extracted radiomics features, LASSO logistic regression was applied to select the most valuable features for the prediction of LN metastasis (**Figures 2A, B**). 22 potential features with non-zero coefficients were selected from the training cohort, including 1 shape and size features, 1 texture features, and 20 wavelet features. ICC ranged from 0.46-0.99 (average=0.95) for all extracted features. The 22 selected radiomics features showed excellent inter-reader agreement, with ICCs > 0.75 (**Table S1**). Rad-score was constructed with the 22 selected features and their coefficients (**Table 2**). Rad-score for each patient was calculated accordingly, patients in positive lymph node metastasis [LNM (+)] group received significant generally higher Rad-score than those in negative lymph node metastasis [LNM (-)] group, which was further confirmed in validation group (**Figure 3**). Univariate and multivariate logistic regression analysis revealed a significant difference between the two groups in rad-score (P < 0.001) (**Table 3**).

**Table 2:** Extracted radiomics features and their coefficients.

Features	Coefficient values
original_shape_Maximum3DDiameter	2.93E-06
original_glcm^a^_Imc2^b^	-1.246123132
wavelet_HLL^c^_glcm^a^_MaximumProbability	0.000126054
wavelet_HLL^c^_glcm^a^_InverseVariance	-0.757235275
wavelet_HLL^c^_glcm^a^_Autocorrelation	1.59E-06
wavelet_HLL^c^_glcm^a^_Imc2^b^	-1.036721409
wavelet_LHL^d^_glrlm^e^_HighGrayLevelRunEmphasis	-0.000188753
wavelet_LHH^f^_firstorder_Uniformity	-5.35E-09
wavelet_LHH^f^_glcm^a^_JointEntropy	0.001410453
wavelet_LHH^f^_glcm^a^_Imc2^b^	-3.233742578
wavelet_LHH^f^_glrlm^e^_LongRunLowGrayLevelEmphasis	1.060581029
wavelet_LLH^g^_firstorder_Median	-0.021394616
wavelet_HLH^h^_glcm^a^_JointEntropy	-0.000723601
wavelet_HLH^i^_glcm^a^_Correlation	2.740702223
wavelet_HHH^j^_firstorder_10Percentile	1.70E-08
wavelet_HHH^j^_glcm^a^_Correlation	1.747207143
wavelet_HHL^k^_firstorder_RobustMeanAbsoluteDeviation	0.000837188
wavelet_HHL^k^_glcm^a^_JointEntropy	0.09227813
wavelet_HHL^k^_glcm^a^_ClusterShade	0.006841175
wavelet_HHL^k^_glcm^a^_Imc2^b^	4.886852754
wavelet_LLL^l^_firstorder_InterquartileRange	0.000397764
wavelet_LLL^l^_glcm^a^_Imc2^b^ LLL_glcm_Imc2	-1.992616499

a: gray-level co-occurrence matrix; b: InformationMeasureCorr2; e: gray level run length matrix; c, d, f, g, h, i, j, k and l represent high-pass filter and lowpass filter on the X, Y, Z three dimensions. The H represents high-pass filter and the L represents low-pass filter. X, Y, Z directions are relative to the standard DICOM LPS (left-posterior-superior) coordinate system.

**Table 3:** Analysis of the potential clinical factors of LN metastasis.

Potential factors	Univariate analysis		Multivariate analysis	
	HR (95%CI)	P	HR (95%CI)	P
**Age**	1.015 (0.971-1.060)	0.513		
**Tobacco use**	1.056 (0.402-2.769)	0.912		
**Alcohol consumption**	1.233 (0.482-3.154)	0.662		
**Location**
Pyriform sinus	1.370 (0.586-3.200)	0.467		
Posterior pharyngeal wall	0.823 (0.342-1.984)	0.665		
Post cricoid	0.465 (0.063-3.445)	0.453		
**MRI-reported LN status**	16.283 (6.033-43.946)	***<0.001***	13.275 (4.180-42.161)	***<0.001***
**Rad-score**	13.137 (4.286-40.266)	***<0.001***	10.945 (3.120-38.399)	***<0.001***

HR, hazard ratio. CI, confidence interval. MRI LN status, Magnetic resonance imaging- reported lymph node status. Bold values are factors with P<0.05 that are significant related with lymph node metastasis in HPSCC, it could be replaced with non-bold font.

### Construction and assessment of radiomics nomogram

**Figure 4:**
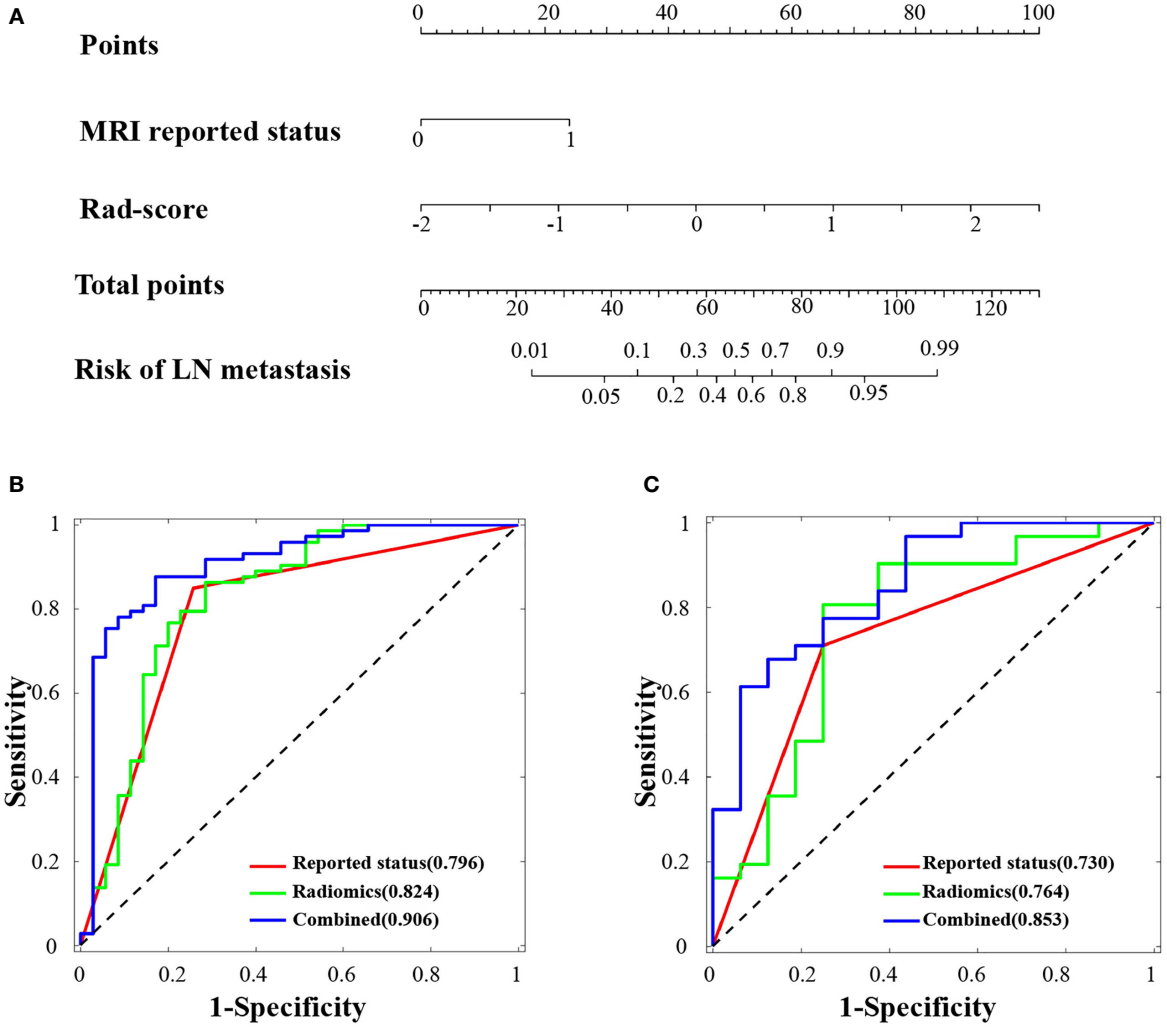
Radiomics nomogram developed and receiver operating characteristic (ROC) curves. **(A)** The radiomics nomogram was developed in the training cohort, with the rad-score and MRI-reported lymph node status incorporated. ROC curves of the combined model (blue lines), radiomics model (green lines) and reported status model (red lines) in the training cohort **(B)** and validation cohort **(C)**.

**Figure 5:**
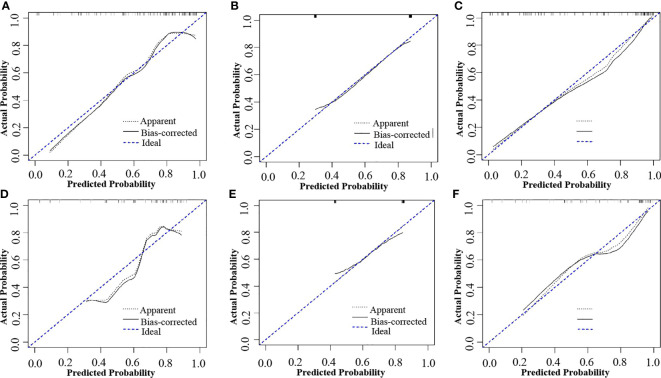
Calibration curves of the combined model, radiomics model and reported status model in the training and validation cohorts, respectively. **(A)** Calibration curve of the radiomics model in the training cohort. **(B)** Calibration curve of the MRI-reported status model in the training cohort. **(C)** Calibration curve of the combined model in the training cohort. **(D)** Calibration curve of the radiomics model in the validation cohort. **(E)** Calibration curve of the MRI-reported status model in the validation cohort. **(F)** Calibration curve of the combined model in the validation cohort. The black dotted line and the black line closer to the blue dotted line indicates a better calibration.

**Figure 6:**
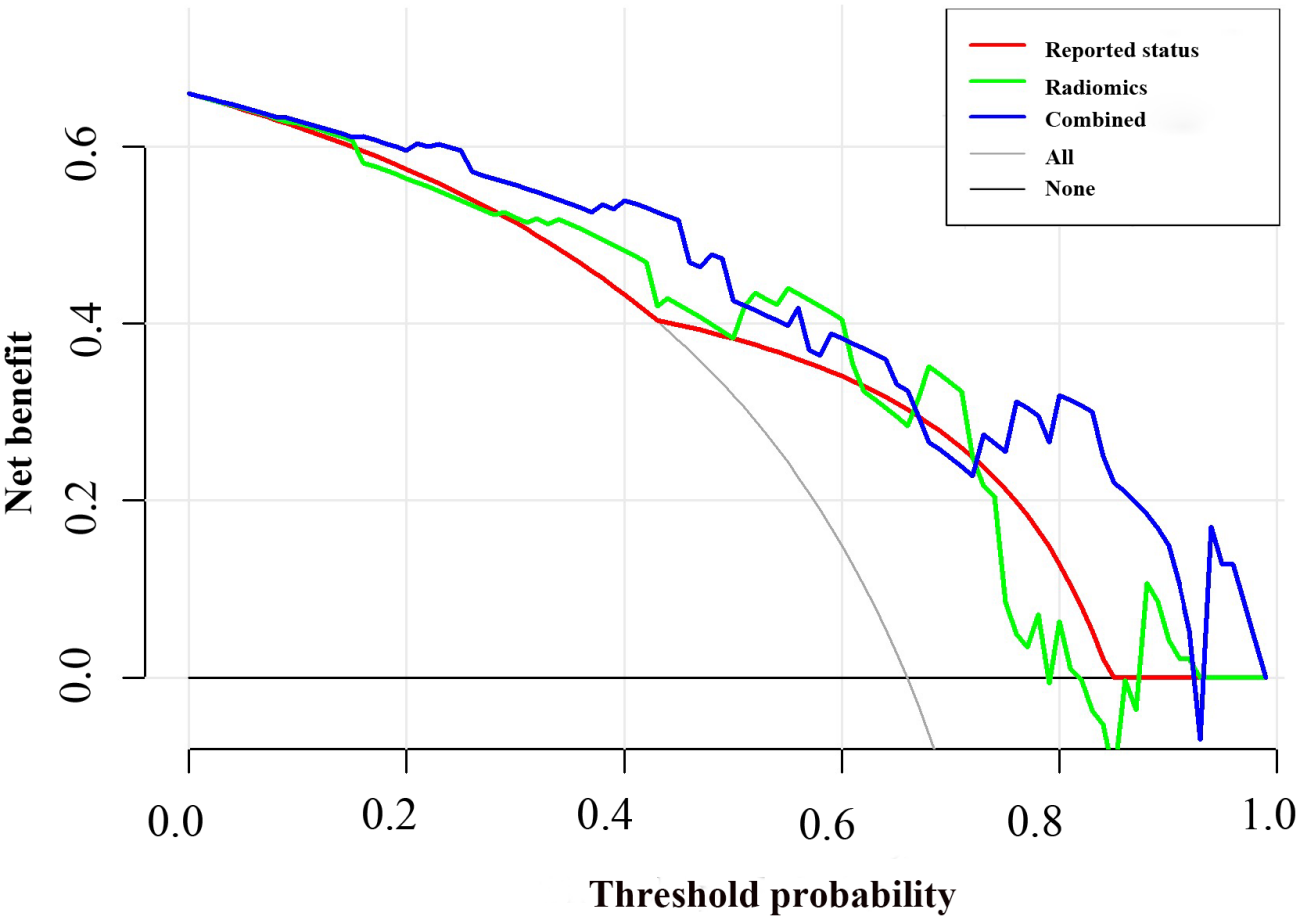
Decision curve analysis for the combined model compared with the radiomics model and reported status alone. The x-axis represented the threshold probability and the y-axis measured the net benefit. The blue line represented the combined model. The green line represented the radiomics model. The red line represented the MRI-reported status model. The grey line represented the assumption that all patients had lymph node (LN) metastasis. The black line represented the hypothesis that no patients had LN metastasis.

The potential parameters were analyzed with univariate and multivariate logistic regression analysis, the Rad-score and MRI-reported LN status were identified as independent risk factors for LN metastasis in HPSCC (**Table 2**). And a visualized nomogram was constructed with selected radiomics features and MRI-reported LN status to predict the individual LN metastasis status (**Figure 4A**). The efficacy of the three models were further compared, our radiomics–clinical model showed superior predictive ability with AUCs of 0.906 (95% CI, 0.840 to 0.972) and 0.853 (95% CI, 0.739 to 0.966) in training and validation groups, respectively (**Figures 4B, C** **)**, which were higher than radiomics model (0.824 and 0.764) and clinical model (0.796 and 0.730). The Delong test showed that the AUC of the combined Model was significantly higher than the clinical model after Bonferroni correction in both training and validation cohorts (both p < 0.0167). However, any other comparisons among the three models, either in training or in validation cohort, showed no significance in terms of AUC by Delong test with Bonferroni correction (all p > 0.0167). The calibration curves of the clinical-radiomics nomogram for the probability of LN metastasis in the training and validation cohorts demonstrated good agreement between prediction and actual observation (**Figure 5**). Furthermore, the decision curve analysis (DCA) suggested that all three models added more benefit when directing treatment decisions compared with treat-none or treat-all scheme in a wide range of threshold probability, indicating the clinical usefulness of these models, and the combined model seemed to added more benefits than the other two models (**Figure 6**).

## Discussion

In the current study, we proposed an MRI-based radiomics nomogram incorporated the radiomic signature and clinical factors for the preoperative prediction of LN metastasis in patients with HPSCC. Our nomogram, incorporated the radiomics signature derived from MRI images and the significant clinical variables, exhibited good discrimination ability in the training cohort (AUC: 0.906) and the validation cohort (AUC: 0.853), outperforming conventional morphology-based diagnostic criteria for LN staging on MRI images, which might provide a straightforward, noninvasive and robust approach for personalized prediction of LN metastasis before surgery.

LN metastasis correlated with poor outcome in patients with HPSCC (19, 20). Due to lack of obvious symptoms, over 60% newly diagnosed patients presented with LN metastasis (21). With advancement of technology, certain lesions (mostly T1 and T2) could be removed with transoral surgeries in HPSCC patients, including transoral laser microsurgery and robotic surgery, which facilitate the tissue healing and functional recovery (22–24). However, neck lymph node metastasis usually needs unilateral/biliteral neck dissection or radiation, which might cause complications such as infection, nerve/vessel injuries, lymphedema and chylous leakage. Patients without LN metastasis could avoid such surgical risk and adverse effects of postoperative radiation (25) if LN status was determined accurately before surgery. Currently, LN status was assessed by radiologist with morphology-based diagnostic criteria that was subjective and experience-dependent, highlighting the significance of preoperative assessment of LN status for individualized therapeutic plan.

Medical Imaging plays a crucial role in assessing LN stage in clinical practice. Nevertheless, it is still challenging by routinely used imaging modalities such as ultrasonography, MRI, CT and PET-CT. The documented sensitivity of PET-CT, CT and MRI in LN metastasis of HPSCC was 50-80% (26–28). While MRI is more widely used and serves as the standard-of-care imaging tool for the preoperative evaluation of LN status, building radiomics models using features extracted from routinely acquired contrast MRI images could be more convenient and efficient. Recent studies have demonstrated the advantages of MRI-based radiomics in assessing LN metastasis in solid tumors (29–32), and achieving an AUC of 0.90 in patients with papillary thyroid carcinoma (30). To the best of our knowledge, our study is the first to established radiomics nomograms for assessing LN metastasis in HPSCC, its stable and accurate predictive performance was also confirmed in training and validation cohort.

During the construction of Rad-score formula, 22 best performing CE-T1WI radiomics features were selected from the 530 radiomics features, among which 20 were wavelet features. Top three of the most closely related features were wavelet_HHL_GLCM_IMC2, wavelet_LHH_GLCM_IMC2 and wavelet-HLH-GLCM-Correlation. The grey-level dependence matrices (GLCM) indicate statistical information about spital distribution of pixel pairs in the image. IMC2 and Correlation describe the complexity and linear dependency of gray level values respectively (33). The wavelet transformation was a multiscale approach to decompose images into high frequency (heterogeneity) and low frequency (homogeneity) for tumor regions (34), while HHL, LHH and HLH filters were known to define regional heterogeneity in intratumor area, and trigger the ability to identify small changes in texture features (35, 36). In total, our findings reflected the advantages of radiomics method to mine high-dimensional information that is difficult to be sensed visually.

There are some limitations to this study. First, this was a retrospective study with data from single center, our finding needs to be validated with further prospective study by larger cohorts from multiple independent centers. Second, we only extracted features from CE-T1WI sequence, while functional images like DCE and DWI might provide valuable information for tissue characterization, hence, future studies should further explore the potential predictive performance of the radiomics model base on multi-sequence. Third, although the probability of cervical lymph node metastasis could be calculated with the formula accordingly in patients with HPSCC, it should also be noted that the ROI in our study was delineated manually, which would be time and labor consuming in clinical practice. Fourth, our nomogram focused on the assessment of LN metastasis status, but not the number and position of the metastatic lymph nodes, which compromised its application in precision therapy, further studies based on the incorporation of primary tumor and suspected lymph nodes might be helpful to discriminate the metastasis status of single lymph node.

## Conclusion

Radiomic features of MRI images are potential biomarkers for LN metastasis in HPSCC. Here, we found our radiomics nomogram that incorporates 22 radiomics features and MRI-reported LN status might be a powerful tool in preoperative prediction of LN metastasis, which would guide the individualized treatment for patients with HPSCC.

## Data availability statement

The raw data supporting the conclusions of this article will be made available by the authors, without undue reservation.

## Ethics statement

The studies involving human participants were reviewed and approved by the Medical Ethics Committee of Xiangya Hospital. The ethics committee waived the requirement of written informed consent for participation.

## Author contributions

SL, HL, JC, and YM: study concept and design, data analysis and interpretation, and drafting of the manuscript. YG, HYL, LT, and PT: substantial contribution to data acquisition and data analysis. SL, YL, DH, YM, and YQ: data interpretation and critical revision of the manuscript. XZ, YL, YM, and YQ: Guarantor of integrity of entire study. All authors: final approval of the manuscript and agreement with all the aspects of the work. All authors contributed to the article and approved the submitted version.

## Funding

This study was supported by the National Natural Science Foundation of China (Nos. 82073009, 81974424, 81874133, 81773243, and 81772903), the National Key Research and Development Program of China (2020YFC1316900 and 2020YFC1316901), the Natural Science Foundation of Hunan Province (Nos. 2019JJ40481 and 2021JJ41012), the Huxiang Young Talent Project (No. 2018RS3024) and Young Scientist Research Fund of Xiangya Hospital (No. 2018Q019).

## Conflict of interest

The authors declare that the research was conducted in the absence of any commercial or financial relationships that could be construed as a potential conflict of interest.

## Publisher’s note

All claims expressed in this article are solely those of the authors and do not necessarily represent those of their affiliated organizations, or those of the publisher, the editors and the reviewers. Any product that may be evaluated in this article, or claim that may be made by its manufacturer, is not guaranteed or endorsed by the publisher.
